# Clover microgreen incorporation in diet‐controlled diabetes and counteracted aflatoxicosis of rats

**DOI:** 10.1002/fsn3.3680

**Published:** 2023-09-15

**Authors:** Tahany A. A. Aly, Sara M. Mohamed, Marwa S. Khattab, Ahmed M. M. Abido, Emam A. Abdel‐Rahim, Ammar Al‐Farga, Frederick Sarpong, Faisal Aqlan

**Affiliations:** ^1^ Regional Center For Food and Feed, Agriculture Research Center Ministry of Agriculture Giza Egypt; ^2^ Pathology Department, Faculty of Veterinary Medicine Cairo University Giza Egypt; ^3^ Biochemistry Department, Faculty of Agriculture Cairo University Giza Egypt; ^4^ Department of Biochemistry, College of Science University of Jeddah Jeddah Saudi Arabia; ^5^ Council for Scientific and Industrial Research‐ Oil Palm Research Institute Kade Ghana; ^6^ Department of Chemistry, College of Sciences Ibb University Ibb Yemen

**Keywords:** aflatoxin, clover, diabetes, histopathology, insulin, phytochemicals

## Abstract

Diabetes mellitus is one of the chronic metabolic diseases whose control remains a challenge. Its increased incidence was mainly attributed to increased environmental contamination. Therefore, this study aims to investigate the effect of feeding clover microgreen (CM) on a diabetes model with or without aflatoxin exposure. Rats were distributed into 8 groups. G1 was a control group. G2 was fed CM. G3 was administered aflatoxin orally. G4 was fed clover and administered aflatoxin. G5 was diabetic rats. G6 was diabetic rats fed CM. G7 was diabetic rats administered aflatoxin. G8 was diabetic rats administered aflatoxin and fed CM. Phytochemical analysis of the CM showed that gardenin was the most common compound. The administration of aflatoxin aggravated diabetes. The groups fed CM showed a decreased glucose concentration compared to the unfed groups. Liver and kidney function parameters were improved by CM. The histopathological alteration of the pancreas, liver, and kidneys was relieved in CM‐fed groups. The area % of insulin in islets of Langerhans was increased in CM‐fed groups. Feeding CM also enhanced the oxidative stress biomarkers. In conclusion, CM improved all evaluated parameters in diabetic rats either exposed to aflatoxin or not compared to the control.

## Graphical abstract

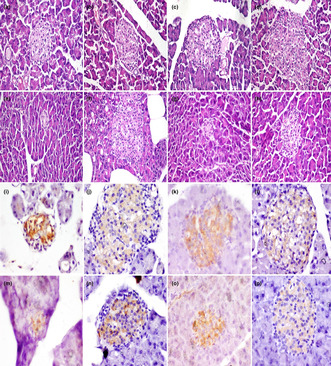

## 1 INTRODUCTION

Type 2 diabetes mellitus is a chronic metabolic disorder. According to the World health organization, 422 million people suffer from diabetes in 2014 with an increased prevalence in low‐ and middle‐income countries than in high‐income countries.

Long‐lasting uncontrolled hyperglycemia in type 2 diabetes affects vital organs such as the kidney, retina, and nervous system, which subsequently affect the quality of life and life expectancy (Garud & Kulkarni, 2018; Pandey et al., 2011). Fatty acid intake induces triglyceride (TG) accumulation in various tissues and insulin resistance in adipocytes. This causes an increase in fatty acid absorption by non‐adipocytes due to the excess transport of proteins and fatty acid binding, adversely affecting insulin‐mediated glucose metabolism. Meanwhile, in the pancreas, prolonged exposure to free fatty acids leads to a recurring vicious cycle in which insulin secretion is impaired by the lipotoxicity mechanism (Lebovitz, 2001). Several factors were investigated previously as contributing factors to the occurrence of diabetes among which was exposure to aflatoxin as a potential risk factor (Akash et al., 2021).

The adverse effects of diabetes can further be aggravated by exposure to environmental contaminants like aflatoxins (Mohamed et al., 2022). Global climate change as evaluated in several studies will have direct and indirect effects on world food demand and increase the susceptibility of crops to future climate (Smith & Gregory, 2013; Wheeler & Von Braun, 2013). The variation in temperature, climate humidity, and precipitation patterns was found to be accompanied by an outbreak of some invasive fungal pathogens (Baazeem et al., 2021). One of the major concerns to food safety is the growth of fungi in cereals with subsequent release of its metabolites as aflatoxins. This could contaminate the entire food chain starting from the livestock products to the end consumers (Kumar et al., 2017). Cell injury due to exposure to aflatoxins is mainly mediated by oxidative stress in addition to damaging DNA, protein, and lipids (Marin & Taranu, 2012). A review article has highlighted the therapeutic effect of phytophenolics as they can reduce oxidative stress, mutagenic, genotoxic, and carcinogenic effects of aflatoxins by regulating the signaling pathways, enhancing the cellular antioxidant balance, modifying the gene expression profile, and alleviating inflammatory responses (Rasouli et al., 2022).

*Trifolium pratense L*. (*Fabaceae*) also known as red clover is a herbaceous plant. Various classes of phytoconstituents have been reported in different parts of the plant including pterocarpans, flavones, phenolic acids, clovamides, and essential oil (Upton et al., 2017). Formononetin, biochanin A, daidzein, and genistein are major isoflavones reported in Trifolium pretense (Wu et al., 2003).

Therefore, this study investigates the combined adverse effect of aflatoxins and diabetes with the possible alleviation using red clover incorporated into a diet.

## 2 MATERIALS AND METHODS

### 2.1 Microgreens of clover

Clover (*Trifolium alexandrium)* (CM) is a member of the *Leguminosae/Fabaceae* family. Its microgreens (CM) were grown in an open field and harvested at the fully expended green cotyledons stage which was 18 days from seed soaking, washing, and hulling (Abdallah, 2008). Harvested CM was air‐dried for 3 days according to a previous study, and ground into powder. Gas chromatography–mass spectrometry (GC/MS) technique was used to assign the phytochemical compounds present in RM powder (Santana et al., 2013) in which GC (Agilent Technology 7890A) associated with a mass selective detector (MSD, Agilent 7000 Triple Quad) having Agilent HP‐5 ms capillary column. The components were identified by comparing their mass spectra with the authentic compounds and by computer matching with the NIST library in addition to comparing the fragmentation pattern of the mass spectral data with those registered in the literature. The analysis was performed in the Regional Center For Food and Feed, Agriculture Research Center, Giza, Egypt.

### 2.2 Aflatoxin preparation

The lyophilized strain of *Aspergillus flavus (NRC, Dokki, Giza, Egypt) was incubated at* 25–29°C for 9 days *on slants of* Czapek's agar media (Davis et al., 1995) and then its spores were transferred on prepared liquid yeast medium and incubated for 9 days at 25–29°C. The media was filtered and the filtrate was kept in bottles wrapped with aluminum foil at 4°C. The concentration of aflatoxin was measured by a slightly modified immunoaffinity method according to the Association of Official Analytic Chemists (AOAC) method (Trucksess et al., 1991). A precalibrated VICAMSeries‐4 fluorometer set was used to measure the concentration at 360 nm excitation and 450 nm emission. Aflatoxin was identified using HPLC equipment with two pumps, column C18, Lichrospher 100 RP‐18 (Agillent 1200 series USA) according to a modified HPLC–AFLATEST procedure.

### 2.3 Animals

Animals purchased from the National Research Center (El Dokki) were housed in plastic cages (3 rats per cage) at 25 ± 2°C and humidity 50%–60%. Rats were acclimated for 2 weeks and had free access to a pelleted diet and water. This study was approved by the Institutional Animal Care and Use Committee, Faculty of Veterinary Medicine, Cairo University (Vet CU01102020224) and was carried out per the guidelines of Care and Use of Laboratory Animals stated by the National Institutes of Health, USPHS.

### 2.4 Induction of type 2 diabetes mellitus

Rats were fed a high‐fat diet for 2 weeks and then injected with a low dose of streptozotocin (single dose of 30 mg kg − 1, i.p.) to induce diabetes type 2 (Zhang et al., 2008). The fasting blood glucose levels of all rats were measured after 7 days of STZ injection. Rats with blood glucose levels ≥200 mg dL − 1 were considered diabetic and were chosen for further experimentation. These rats were maintained on HFD until the termination of the study.

### 2.5 Diets and their preparation

Four different diets were prepared and manufactured into pellets. A control diet was based on the AIN‐76: a CM diet in which 10% CM powder replaced corn starch, a high‐fat diet with 20% palm oil, a high‐fat and CM diet with 20% palm oil, and 10% CM (Table 1).

**TABLE 1:** Composition of diets.

Ingredients	Control diet	CM diet	High‐fat diet	High‐fat and CM diet
Casein	20.0	20.0	20.0	20.0
Corn starch	65.0	55.0	50.0	40.0
Mineral Mix^2^	3.5	3.5	3.5	3.5
Vitamin mix^2^	1.0	1.0	1.0	1.0
DL‐Methionine	0.3	0.3	0.3	0.3
Choline bitartrate	0.2	0.2	0.2	0.2
Cellulose powder	5.0	5.0	5.0	5.0
Palm oil	5.0	5.0	20.0	20.0
Clover microgreen	–	10.0	–	10.0

*Note*: 1, weight percentage; 2, based on AIN‐76.

### 2.6 Chemicals

STZ and DMSO were obtained from Sigma‐Aldrich (art no. 7029.1) (Merck KGaA) while kits for insulin hormone and liver profile were products of Thermo Fisher Scientific, Waltham, MA, and Biodiagnostic, respectively.

### 2.7 Experimental design

**FIGURE 1:**
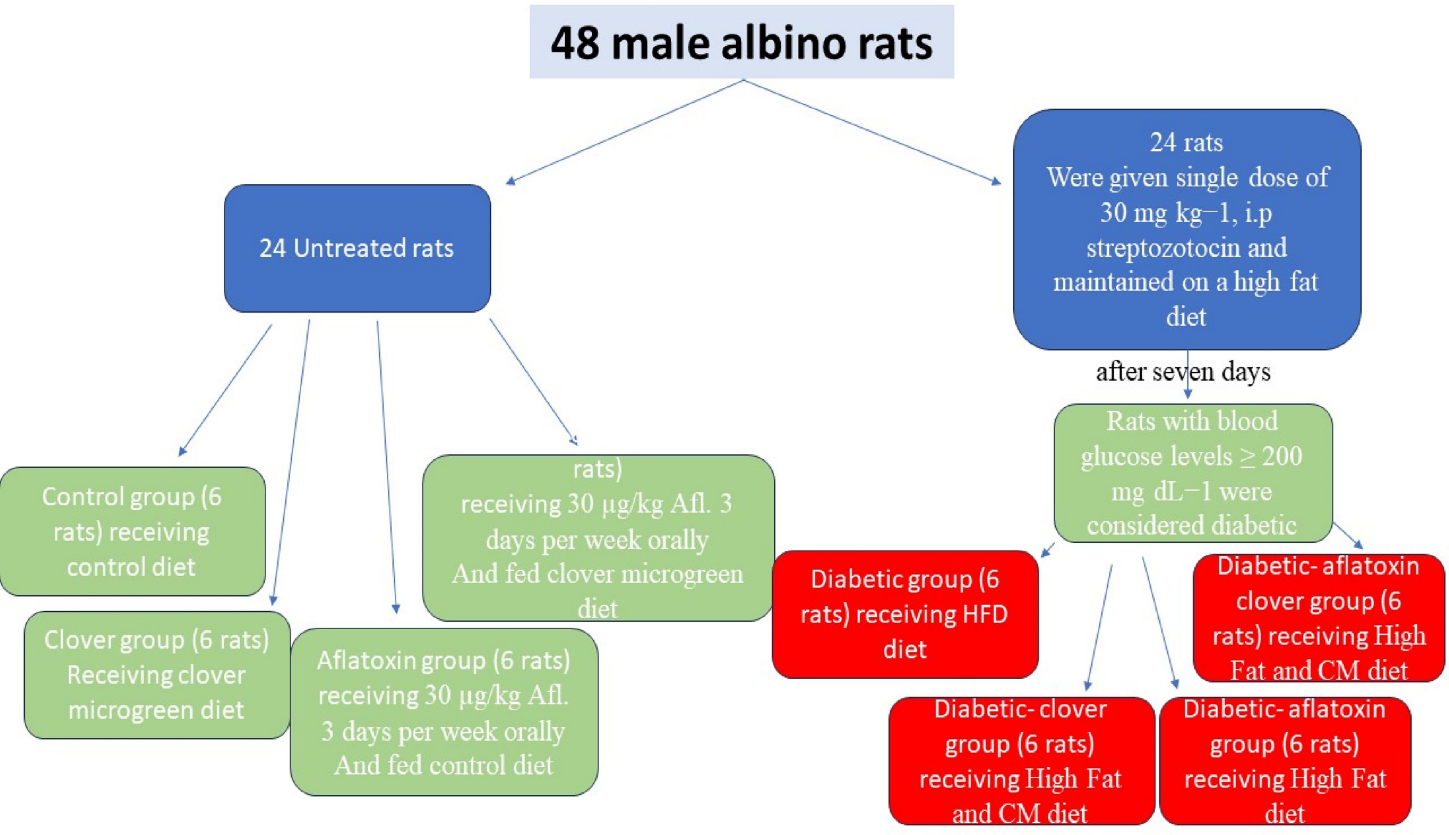
Flowchart showing the experimental design.

Forty‐eight male albino rats were distributed into 8 groups (6 rats each). G1 rats were non‐diabetic and fed the control diet. G2 rats were non‐diabetic and fed a CM diet. G3 rats were non‐diabetic, fed the control diet, and orally given aflatoxin (30 μg/kg) 3 days per week (Mahato et al., 2019). G4 rats were non‐diabetic, administered aflatoxin, and fed a CM diet. G5 rats were diabetic and fed a high‐fat diet (HFD). G6 rats were diabetic and fed a high‐fat CM diet. G7 rats were diabetic, fed HFD, and administered aflatoxin. G8 rats were diabetic, fed a high‐fat CM diet, and administered aflatoxin (Figure 1). The initial and final weights of rats were recorded. Bodyweight (BW) = final weight‐initial weight. Bodyweight gain was calculated by the following equation: BWG/100 g = BW/initial weight × 100 (Novelli et al., 2007). The experiment ended after 6 weeks.

### 2.8 Determination of glucose level in plasma

The enzymatic and calorimetric method was used to estimate the glucose level in blood plasma (Trinder, 1969) in which the oxidation of glucose to gluconic acid was catalyzed by glucose oxidase (GOD).

### 2.9 Determination of serum insulin hormone

The serum insulin level in rats was estimated by an ELISA kit (Thermo Fisher Scientific) following the manufacturer's protocol (Temple et al., 1992). The plate was read by an ELISA reader at 450 nm. The absorbance values of standard insulin concentrations were used to make a standard curve on a log–log paper. The insulin concentration of the samples was calculated from the standard curve. The following equations were used to calculate the homeostatic model assessment of Beta cells function (HOMA‐B), homeostatic model assessment of insulin resistance (HOMA‐IR), homeostatic model assessment of insulin sensitivity (HOMA‐% S), and disposition index (DI).

$$\begin{array}{c} \text{HOMA}-\%B=\left[20\times \text{fasting insulin}\;\left(\mathrm{mU}/L\right)\right]/\\ \;\left[\left(\text{fasting glucose}\;\left(\mathrm{mg}/\mathrm{dL}\right)\times 0.055\right)–3.5\right]. \end{array}$$

$$\begin{array}{c} \text{HOMA}-\mathrm{IR}=[\text{fasting insulin}\;\left(\mathrm{mU}/L\right)\\ \;\times \text{fasting glucose}\;\left(\mathrm{mg}/\mathrm{dl}\right)\times 0.0555]/22.5. \end{array}$$

$$\text{HOMA}-\%S=\left[1/\text{HOMA}-\mathrm{IR}\right]\times 100.$$

$$\text{Disposition index}\;1=\left(\text{HOMA}-\%S/100\right)\times \left(\text{HOMA}-\%B/100\right).$$

### 2.10 Serum biochemistry parameters

The collected sera were used in the assessment of total bilirubin and total protein concentrations, AST, ALT, and ALP activity following previous methods (Belfield & Goldberg, 1971; Gornnall et al., 1949; Reitman & Frankel, 1957; Walter & Gerarde, 1970). Serum urea and creatinine concentrations and serum lactate dehydrogenase activity (LDH) were measured based on previous methods (Naithani et al., 2006; Fawcett & Scott, 1960; Moore & Sharer, 2017).

### 2.11 Histopathology

Pancreas, liver, and kidney tissue specimens of rats were fixed in 10% neutral buffered formalin, processed by ascending concentration of ethanol and xylene, embedded in paraffin wax, and sectioned into 4‐μm‐thick sections. Tissue sections were stained by hematoxylin and eosin stain, examined by light microscopy, and photographed using a digital camera (Olympus XC30).

### 2.12 Immunohistochemistry

Primary antibodies against insulin were used to detect insulin in paraffin‐embedded tissue sections of the pancreas (Invitrogen, Thermo‐Fisher Scientific) after antigen retrieval using citrate buffer PH6. The avidin‐biotin‐peroxidase complex kit (Dako, North America, Inc.) was used to detect the primary antibody. The brown color was developed using 3,3′‐Diaminobenzidine as a substrate. Image analysis of pancreas photographs using Image J software was used to measure the area % of positive insulin in beta cells of the pancreatic islets in three photos/rats in each group at a 400× magnification power.

### 2.13 Determination of oxidative stress in liver

Oxidative stress biomarkers were assessed in liver homogenate. Superoxide dismutase (SOD) activity was determined according to Nishikimi et al. (1972) method. Superoxide dismutase‘s (SODs) enzymes are metalloenzymes that catalyze the dismutase of the superoxide anion to molecular oxygen and hydrogen peroxide defense mechanism. This relies on the ability of the enzyme to inhibit the phenazine methosulfate‐mediated reduction of nitro blue tetrazolium.

Reduced glutathione (GSH) was determined based on the reduction of 5.5 dithiobis (2‐nitrobezoic acid) (DTNB) with glutathione (GSH) to produce a yellow compound. The colored chromogen was directly proportional to GSH concentration which can be measured at 405 nm by its absorbance.

Catalase (CAT) activity was estimated by measuring the breakdown of hydrogen peroxide in the reaction mixture. The reaction was started by the addition of 50 1 of suitably diluted supernatant to 3 mL of phosphate buffer H_2_O_2_ solution. Initial absorbance at 240 nm was read after 20 s against a reference cuvette in which the same amount of distilled water was added instead of H_2_O_2_. The time required for initial absorbance to decrease by 0.05 units was noted. Catalase activity in the sample was calculated and expressed in U/mg of protein (Cohen et al., 1970).

Malondialdehyde (MDA) in an acidic medium at a temperature of 95°C for 30 min reacted to form a reactive product of thiobarbituric acid where the absorbance of the resultant pink product can be determined at 534 nm (Aebi, 1984; Beutler et al., 1963; Nishikimi et al., 1972).

### 2.14 Statistical analysis

Statistical analysis of obtained data was analyzed using SPSS software. The homogeneity of variances was tested then the ANOVA test was used to detect significance followed by suitable post hoc tests. The data presented was the mean value ± standard error (Kinnear & Gray, 1999; Waller & Duncan, 1969).

## 3 RESULTS AND DISCUSSION

### 3.1 Phytochemical content of CM

Egyptian clover microgreen was rich in Gardenin (29.11%) followed by 5,7‐Dimethoxy‐4‐methyl coumarin (15.96%) belonging to the large subclass of benzopyrones and having a structure of fused benzene and a‐pyrone ring. Other compounds found include 2‐Hexadecanol (11.15%), 1‐Dodecanol, 3,7,11‐trimethyl (10.3%), 9‐cis‐Retinoic acid (8.59%) (Table 2).

**TABLE 2:** Phytochemical compounds identified in the ethanolic extract fractionation of Egyptian clover microgreen by GC–MS.

No.	R.T	Compound name	Area sum %
1	13.73	2,6‐Octadiene, 2,6‐dimethyl‐	2.28
2	13.83	Valeranone (+)‐	0.4
3	13.90	Isophytol	0.9
4	14.04	Retinyl propionate	1.78
5	14.58	Phytosphingosine	4.87
6	14.90	2‐Hexadecanol	11.15
7	15.71	1‐Dodecanol, 3,7,11‐trimethyl‐	10.3
8	16.09	9‐cis‐Retinoic acid	8.59
9	16.72	2‐Pentadecanone, 6,10,14‐trimethyl‐	0.39
10	17.19	Phytol	1.56
11	17.25	5,7‐Dimethoxy‐4‐methylcoumarin	15.96
12	17.86	Geranyl isovalerate	1.31
13	18.06	3‐Hydroxy‐7,8,2′‐trimethoxyflavone	0.72
14	18.40	3‐(3,4‐Dimethoxyphenyl)‐7‐methyl‐4phenylcoumarin	0.88
15	18.76	Vitexin	0.37
16	18.89	Isovitexin	0.89
17	18.96	Phytanic acid	5.82
18	19.61	2’‐Hydroxy‐2,4,4′,5‐tetramethoxychalcone	0.62
19	19.74	2’‐Hydroxy‐3,4,5‐trimethoxychalcone	0.42
20	19.96	3,6,3′,4’‐Tetramethoxyflavone	0.62
21	20.34	Gardenin	29.11
22	20.86	3‐(3,4‐Dimethoxyphenyl)‐4‐methylcoumarin	0.52
23	21.22	7,3′,4′,5’‐Tetramethoxyflavanone	0.53

### 3.2 Bodyweight gain

The rats' body weight gain (BWG) was decreased in G5‐8, especially G7, compared to G1. The BWG was improved relatively in G8 (STZ‐Aflatoxin‐CM) compared to G7. G2 receiving CM diet recorded the highest BWG (Table 3).

**TABLE 3:** Body weight gain of rats at the end of the 6‐week experimental period.

Treatment	Initial weight g	Final weight G	(B.W) g	BWG/100 g
G1 control	118.8 ± 8.91^c^	200.57 ± 10.94^a^	81.77 ± 25.37	68.83
G2 CM	123.97 ± 3.47^b^	211.96 ± 3.21^a^	87.99 ± 430	70.98
G3 aflatoxin	124.40 ± 14.09^b^	168.78 ± 6.35^b^	44.39 ± 31.69	35.68
G4 aflatoxin, CM	126.32 ± 6.27^b^	186.36 ± 7.84^b^	60.04 ± 3.80	47.53
G5 STZ	124.80 ± 14.39^b^	132.27 ± 2.77^c^	7.47 ± 13.77	5.99
G6 STZ, CM	116.62 ± 12.53^c^	126.12 ± 8.95^c^	9.50 ± 12.41	8.15
G7 STZ, aflatoxin	135.73 ± 18.69^a^	93.33 ± 17.68^d^	−42.41 ± 19.40	−31.25
G8 STZ, aflatoxin, CM	111.43 ± 6.66^c^	116.06 ± 7.33^d^	4.63 ± 6.29	4.16

Abbreviation: BW = bodyweight. BWG = body weight gain. CM = clover microgreen.

*Note*: All values are represented as mean ± S.D. Means with different letters superscript are significantly different (*p* < .05).

### 3.3 Blood glucose and insulin level

The glucose level of rats was elevated significantly in G3–G8 fed with aflatoxin and/or injected with STZ compared to G1 and G2. G7 recorded the highest glucose level. The glucose level however was lowered in diabetic and/or aflatoxicated groups fed CM compared to their counterparts.

The insulin level was significantly lower in G5‐G8 with the lowest decrease in G7 in comparison to the G1 and G2 groups (Table 4).

**TABLE 4:** Blood glucose and insulin levels in different experimental animal groups at the end of the experimental period.

Groups	Treatment	Blood glucose		Blood insulin	
		mg/dL	m mol/dL	mu/L	ng/L
G1	Control	100 ± 8.1^e^	5.56 ± 0.45^e^	7.37 ± 0.44^a^	1.84 ± 0.08^a^
G2	CM	101 ± 9.2^e^	5.61 ± 0.51^e^	7.70 ± 0.52^a^	1.93 ± 0.10^a^
G3	Aflatoxin	131 ± 11.1^c^	7.28 ± 0.62^c^	6.67 ± 0.42^ab^	1.67 ± 0.08^ab^
G4	Aflatoxin, CM	116 ± 10.3^d^	6.44 ± 0.57^d^	7.00 ± 0.54^ab^	1.75 ± 0.08^ab^
G5	STZ	150 ± 8.2^b^	8.33 ± 0.46^b^	6.20 ± 0.41^b^	1.55 ± 0.08^b^
G6	STZ, CM	124 ± 8.4^cd^	6.89 ± 0.47^cd^	6.37 ± 0.43^b^	1.59 ± 0.08^b^
G7	STZ, aflatoxin	176 ± 8.3^a^	9.78 ± 0.46^a^	5.70 ± 0.39^b^	1.43 ± 0.09^b^
G8	STZ, aflatoxin, CM	151 ± 10.1^b^	8.39 ± 0.56^b^	5.93 ± 0.41^b^	1.48 ± 0.08^b^

Abbreviation: CM = clover microgreen.

*Note*: All values are represented as mean ± SD. Means with different letters are significantly different (*p* < .05).

### 3.4 Homeostatic model assessment

The Beta cells function significantly declined in G3–G8 with the lowest value in the G7 group. Feeding CM in G4, G6, G8 significantly improved the Beta cells function compared to G3, G5, and G7, respectively.

However, insulin resistance was significantly high in G3, G5, and G7 groups compared to G1 and G2. However, in G4, and G6 groups fed CM, it was decreased compared to their counterparts and was insignificantly similar to G1 and G2.

Insulin sensitivity was significantly reduced in G5 and greatly in G7 compared to G1 and G2. On the contrary, it was enhanced in G6 and G8 groups fed CM and was comparable with the control.

The deposition index was significantly reduced in G3–G8 especially in G7 compared to G1 and G2. Nevertheless, G4, G6, and G8‐fed CM recorded a significantly improved deposition index compared to their counterparts (Table 5).

**TABLE 5:** Homeostatic model assessment of Beta cells function (HOMA‐B), homeostatic model assessment insulin resistance (HOMA‐IR), homeostatic model assessment insulin sensitivity (HOMA‐% S), and disposition index (DI) of the experimental rats.

Groups	Treatment	HOMA‐B	HOMA‐IR	HOMA‐%S	Disposition index
		Value	Value	Value	Value
G1	Control	71.55 ± 6.61^a^	1.82 ± 0.12^c^	54.95 ± 4.41^a^	0.393 ± 0.031^a^
G2	CM	72.99 ± 6.87^a^	1.92 ± 0.16^c^	52.08 ± 5.01^a^	0.380 ± 0.029^a^
G3	Aflatoxin	35.29 ± 3.12^c^	2.16 ± 0.20^b^	47.30 ± 4.18^ab^	0.167 ± 0.011^c^
G4	Aflatoxin CM	47.62 ± 4.01^b^	2.00 ± 0.14^bc^	50.00 ± 4.27^a^	0.238 ± 0.020^b^
G5	STZ	25.67 ± 2.41^d^	2.30 ± 0.19^b^	43.48 ± 4.34^b^	0.112 ± 0.010^d^
G6	STZ, CM	37.58 ± 3.14^c^	1.95 ± 0.17^c^	51.28 ± 5.01^a^	0.193 ± 0.017^c^
G7	STZ, aflatoxin	18.15 ± 1.12^e^	2.48 ± 0.21^a^	40.32 ± 3.98^b^	0.073 ± 0.006^e^
G8	STZ, aflatoxin, CM	24.25 ± 2.21^d^	2.21 ± 0.21^b^	45.25 ± 4.26^ab^	0.110 ± 0.009^d^

Abbreviation: CM = clover microgreen.

*Note*: All values are represented as mean ± SD. Means with different letters are significantly different (*p* < .05).

### 3.5 Liver function parameters

Rats in G3–G8 had significantly elevated liver function enzymes and total bilirubin and a significantly decreased total protein compared to G1 and G2. The liver function parameters were the worst in G7. On the reverse, G4, G6, and G8‐fed CM recorded improved liver function parameters except for the total protein which still did not record a significance compared to their counterparts (Table 6).

**TABLE 6:** Liver function enzymes, total bilirubin, and total protein in different groups.

Treatment	AST U/L	ALT U/L	ALP U/L	Bilirubin total mg/dL	T.Protein g/dL
G1 control	124.83 ± 10.12^e^	64.83 ± 5.77^c^	722.50 ± 20.26^d^	0.51 ± 0.07d	6.86 ± 0.09^a^
G2 CM	119.99 ± 8.71^e^	66.50 ± 4.61^c^	727.33 ± 50.41^d^	0.67 ± 0.11c	7.22 ± 0.19^a^
G3 aflatoxin	247.67 ± 9.29^ab^	93.17 ± 5.84^b^	1268.67 ± 101.54^a^	0.71 ± 0.09b	6.43 ± 0.13^b^
G4 aflatoxin CM	167.00 ± 2.00^c^	80.00 ± 1.00^b^	903.00 ± 79.70^b^	0.65 ± 0.23c	6.38 ± 0.12^b^
G5 STZ	189.50 ± 6.06^b^	71.83 ± 2.84^bc^	980.50 ± 17.50^b^	0.77 ± 0.16ab	6.13 ± 0.28^b^
G6 STZ,clover	160.00 ± 2.78^c^	60.83 ± 2.36^c^	933.67 ± 129.96^b^	0.56 ± 0.07^c^	6.55 ± 0.48^ab^
G7 STZ, aflatoxin	322.00 ± 12.12^a^	125.00 ± 6.06^a^	1303.67 ± 117.27^a^	0.89 ± 0.02^a^	5.86 ± 0.95^b^
G8 STZ,aflatoxin, CM	179.67 ± 2.84^c^	81.83 ± 2.25^b^	964.67 ± 81.29^b^	0.60 ± 0.09^c^	6.14 ± 0.47^b^

*Note*: All values are represented as mean ± SD. Means with different letters are significantly different (*p* < .05).

### 3.6 Kidney function parameters and LDH activity

LDH activity was increased significantly in G3–G8, with the highest elevation in G7 compared to control; however, it was relatively decreased in G4, G6 and G8 compared to their counterparts. The urea concentration was significantly increased in G3 only compared to G1. The creatinine concentration was significantly elevated in G3, G5, G6, G7, and G8 (Table 7).

**TABLE 7:** Kidney function enzymes and lactate dehydrogenase (LDH) in different groups.

Treatment	LDH activity U/L	Urea mg/dL	Creatinine mg/dL
G1 control	1388.50 ± 29.50^e^	39.67 ± 1.44^b^	0.61 ± 0.11^c^
G2 CM	1355.67 ± 75.01^e^	42.50 ± 3.26^b^	0.62 ± 0.09^c^
G3 aflatoxin	2136.67 ± 91.12^b^	52.17 ± 9.17^a^	0.81 ± 0.19^a^
G4 aflatoxin, CM	1805.67 ± 20.84^c^	36.50 ± 3.07^b^	0.69 ± 0.18^c^
G5 STZ	1743.17 ± 58.31^c^	40.67 ± 2.13^b^	0.71 ± 0.08^b^
G6 STZ. CM	1478.50 ± 26.30^d^	33.83 ± 3.80^b^	0.77 ± 0.05^b^
G7 STZ, aflatoxin	2426.00 ± 77.74^a^	41.00 ± 2.00^b^	0.80 ± 0.06^a^
G8 STZ, aflatoxin, CM	1435.67 ± 42.01^d^	37.67 ± 4.93^b^	0.87 ± 0.20^a^

*Note*: All values are represented as mean ± SD. Means with different letters are significantly different (*p* < .05).

### 3.7 Histopathological and immunohistochemical findings in pancreas

**FIGURE 2:**
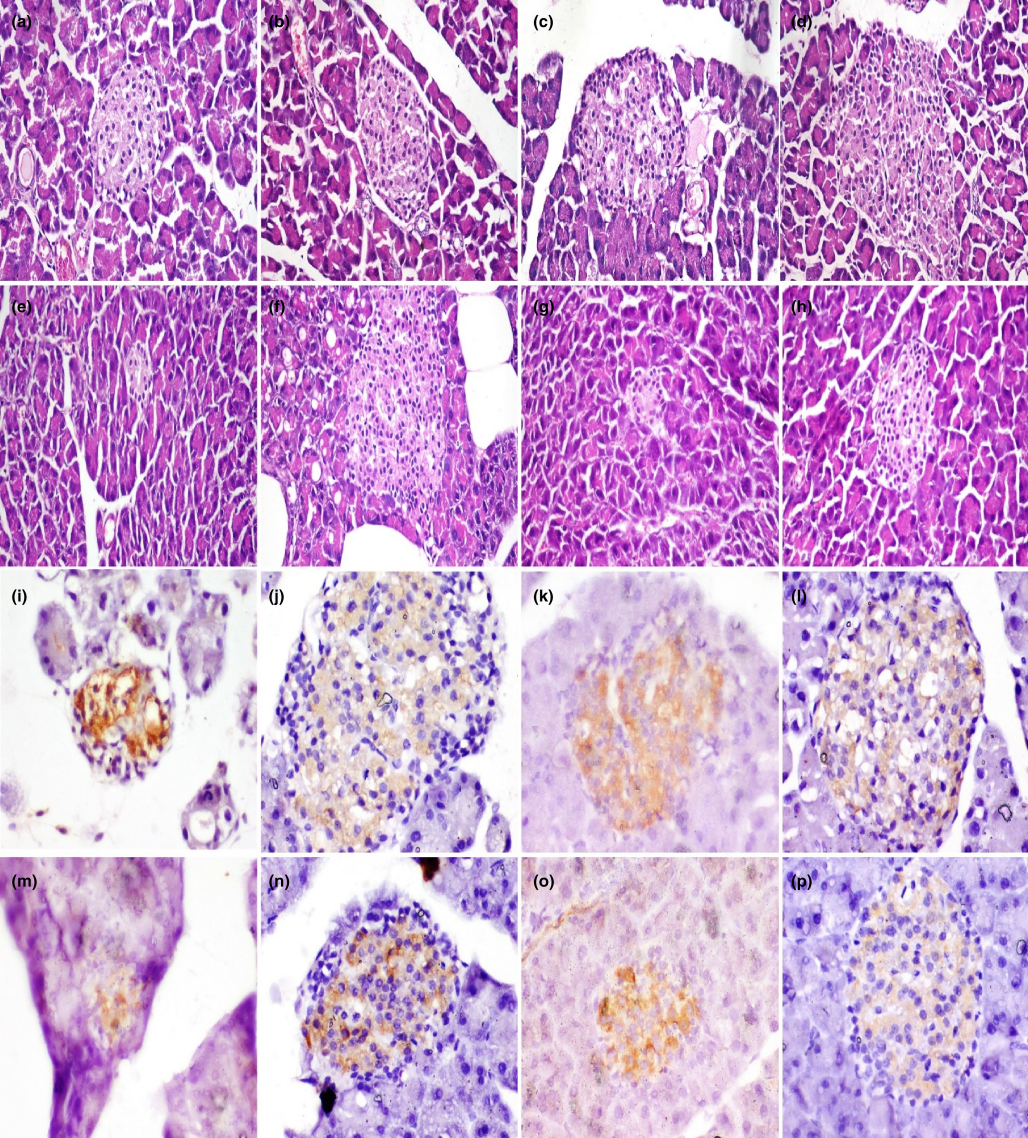
Pancreas of rats showing islets of Langerhans. (a) Normal histological structure of pancreas in control group, and (b) CM group. (c) Moderate size with irregular boundaries and degenerated cells in aflatoxin group, (d) the islets of Langerhans were large and had few degenerated cells in aflatoxin and CM group, (e) the islets of Langerhans were small in size, exhibited fibrosis and vacuolation of cells in the STZ group, (f) moderate sized islets in STZ and CM group, (g) islets of Langerhans had irregular boundaries and degenerated cells in STZ and aflatoxin group, (h) moderate sized islets in STZ, aflatoxin, and CM group. Hematoxylin and eosin stain (X200). (i–p). Immunohistochemistry of insulin‐positive beta cells in islets of Langerhans of rat pancreas in (i) many positive cells in islets of control group, (j) and CM group, (k) moderate positive cells in islets of aflatoxin group, (l) increased positive cells in islets of aflatoxin and CM group, (m) few positive cells in islets of STZ group, (n) moderate positive cells in islets of STZ and CM group, (o) few positive cells in islets of STZ and aflatoxin group, (p) moderate positive cells in islets of STZ, aflatoxin, and CM. Immunoperoxidase stain (X400).

Histopathological examination of the pancreas in the control and clover groups showed normal structure (Figure 2a, b). In the aflatoxin group (G3), the islets of Langerhans were of moderate size with irregular boundaries and degenerated cells (Figure 2c). In G4, the islets of Langerhans were larger than the previous group and had fewer degenerated cells (Figure 2d). In G5, the islets of Langerhans were small in size and exhibited fibrosis and vacuolation of cells (Figure 2e). In G6, the lesions recorded in the islets of Langerhans were less recorded in this group than in the previous one (Figure 2f). In G7, the microscopy of islets of Langerhans revealed irregular boundaries and degenerated cells (Figure 2g) however in G8, these lesions were less observed (Figure 2h).

**FIGURE 3:**
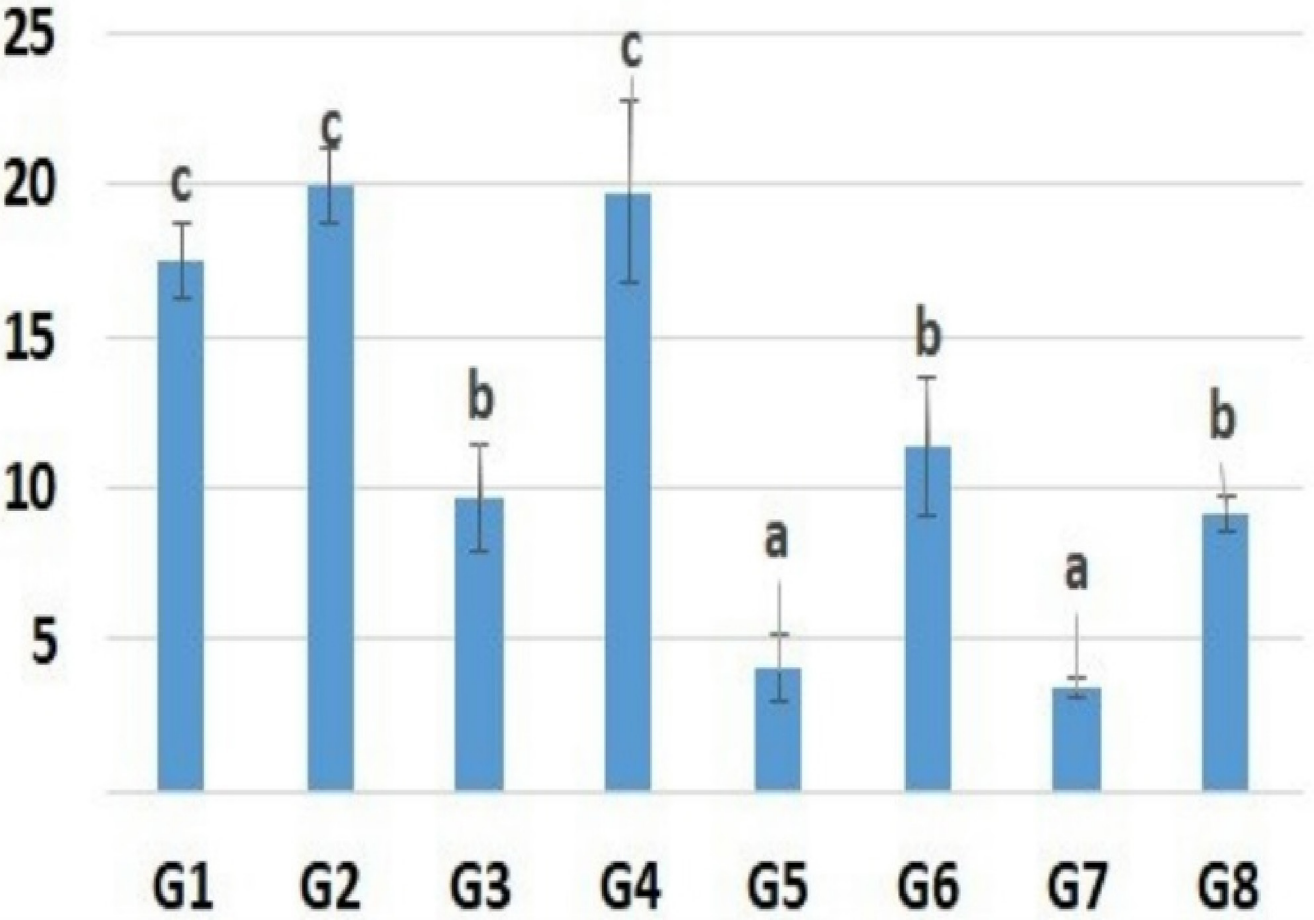
Area percent of Insulin immunohistochemistry in different groups. Data are presented as mean value ± standard error. Columns bearing different lowercase letters are considered significant at *p* value <.05.

The islets of Langerhans in the pancreas showed insulin‐positive cells arranged in a regular continuous cord in G1 and G2. In G3 (Aflatoxin group), the area % of insulin‐positive beta cells moderately decreased and the cells were irregularly arranged compared to the control group. In G4, the area % of beta cells were comparable to that of the control group and cells were well arranged. The area % of beta cells in the pancreas of G5 was greatly reduced compared to those of G1. In G6, the area % of beta cells increased significantly compared to that of G5. In G7, the area % of insulin‐positive beta cells recorded the lowest value of all groups. However, in G8, the area % of insulin‐positive beta cells was partially restored (Figure 3).

**FIGURE 4:**
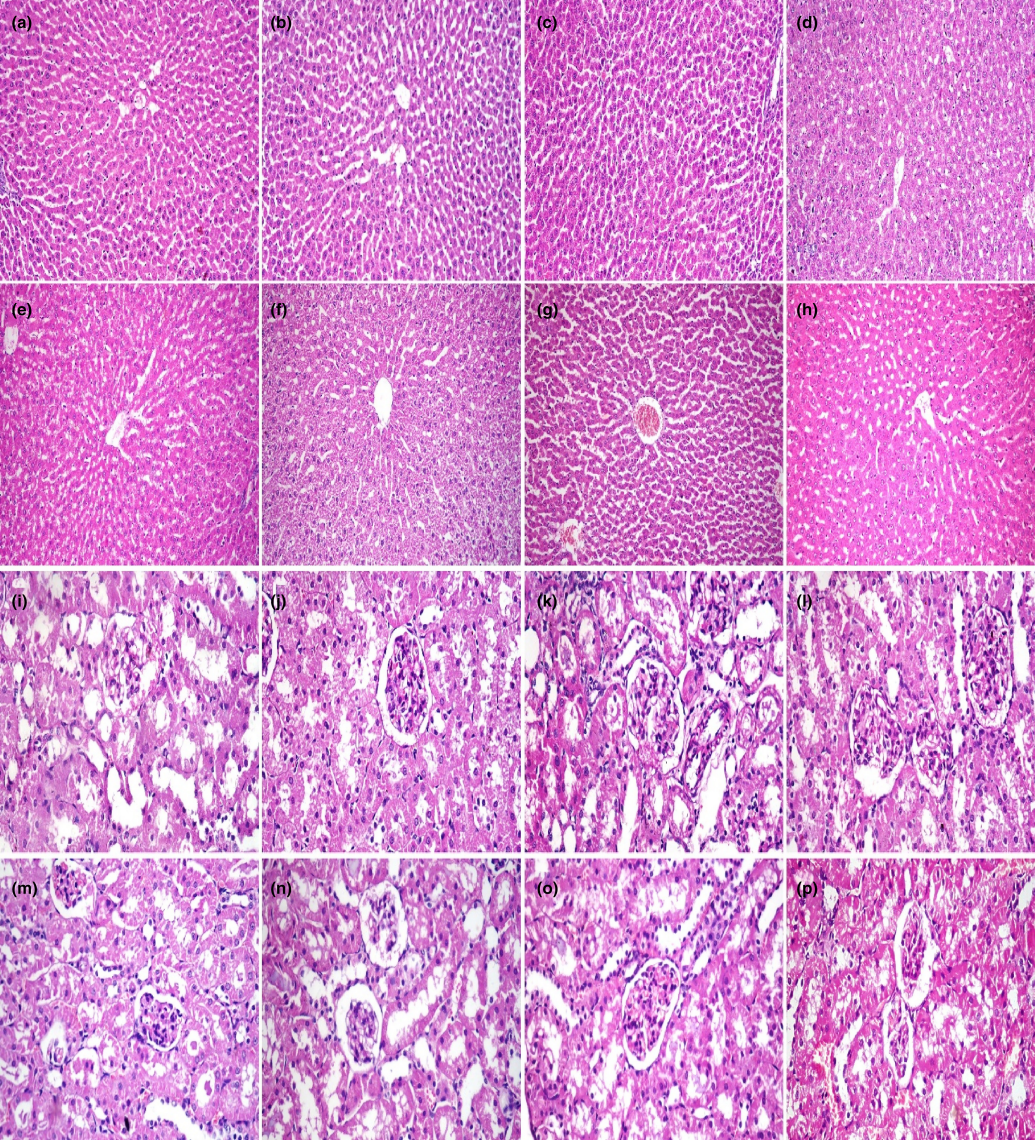
Histopathology of liver and kidneys. (a–h) Histopathological structure of the liver of rats. (a) Normal histological structure of liver in control group, and (b) CM group, (c) hepatocytes were hypertrophied in the aflatoxin group, (d) aflatoxin and CM group, (e) vacuolation of periportal hepatocytes in the STZ group, (f) mild histological alteration in the STZ and CM group, (g) vacuolation of periportal hepatocytes in the STZ and aflatoxin group, (h) moderate histological alteration in STZ, aflatoxin, and CM (X100). (i–p) Kidney of rats in different groups. (i) Normal histological structure of kidney in control group and, (j) CM group, (k) the tubular epithelium was hypertrophied and showed vacuolar degeneration in aflatoxin group, (l) mild histopathological alteration in aflatoxin and CM group, (m) the glomeruli exhibited increased glomerular matrix, mesangial cell hyperplasia, and mild thickening of the glomerular basement membrane in the STZ group, (n) mild histopathological alteration in the STZ and CM group, (o) the glomeruli exhibited increased glomerular matrix, mesangial cell hyperplasia, and mild thickening of the glomerular basement membrane in the STZ and aflatoxin group, (p) moderate histopathological alteration in STZ, aflatoxin, and CM. Hematoxylin and eosin stain (X 200).

Microscopy of the liver in the G1 and G2 revealed normal histological structure. In G3, the hepatocytes were hypertrophied with binucleation, karyomegaly, and mild solitary necrosis. In G4, the histopathological alteration of the liver was less severe compared to G3. The lesion observed in G5 and G7 was vacuolation of periportal hepatocytes. However, this lesion was less observed in G6 and G8 (Figure 4a–h).

Microscopy of the kidneys in G1 and G2 revealed a normal histological structure, whereas in G3, the tubular epithelium was hypertrophied and showed vacuolar degeneration. In G4, the kidney microscopy revealed mild histopathological alteration. In G5, the glomeruli exhibited increased glomerular matrix, mesangial cell hyperplasia, and mild thickening of the glomerular basement membrane in addition to tubular cell degeneration and necrosis with luminal casts. In G7, similar lesions were observed but were more severe compared to the previous group. In G6 and G8, these lesions were reduced compared to G5 and G8 (Figure 4i–p).

### 3.8 Oxidative stress biomarkers in liver

The MDA content was increased significantly in G3–G8 compared to G1 and G2. However, it was significantly reduced in G4, G6, and G8‐fed CM compared to G3, G5, and G7, respectively.

G3–G8 rats had a significantly decreased concentration of GSH content and activity of antioxidant enzymes compared to G1 and G2. Contrarywise, G4, G6, and G8 rats had significantly improved GSH content and antioxidant enzyme activities compared to G3, G5, and G7 (Table 8).

**TABLE 8:** Effect of clover microgreen on liver tissue peroxidation of rats.

Treatment	MDA content nmol/g	Liver oxidation system			
		GSH content (μmol/mL)	SOD activity (U/g)	CAT activity (U/g)	GST activity (μmol/gt)
G1 control	6.361 ± 0.421^de^	0.411 ± 0.032^a^	77.10 ± 8.88^a^	158.11 ± 7.81^a^	6.13 ± 0.43^a^
G2 clover	5.870 ± 0.362^e^	0.421 ± 0.031^a^	79.13 ± 4.44^a^	162.20 ± 8.77^a^	6.30 ± 0.48^a^
G3 aflatoxin	10.112 ± 0.712^b^	0.222 ± 0.013^d^	47.04 ± 3.71^d^	99.12 ± 6.16^cd^	4.11 ± 0.21^d^
G4 aflatoxin, CM	8.111 ± 0.532^c^	0.297 ± 0.016^c^	55.16 ± 3.21^c^	111.22 ± 7.12^c^	5.00 ± 0.31^b^
G5 STZ	8.221 ± 0.521^c^	0.301 ± 0.020^c^	60.09 ± 4.02^c^	120.12 ± 8.27^bc^	5.07 ± 0.32^b^
G6 STZ, CM	6.842 ± 0.421^d^	0.402 ± 0.020^b^	69.12 ± 4.26^b^	136.08 ± 8.01^b^	6.00 ± 0.46^a^
G7 STZ, aflatoxin	12.781 ± 0.941^a^	0.154 ± 0.010^e^	35.03 ± 2.12^e^	84.20 ± 5.14^e^	3.45 ± 0.21^e^
G8 STZ, aflatoxin, CM	9.872 ± 0.542^b^	0.213 ± 0.017^d^	45.16 ± 2.56^d^	96.09 ± 5.51^d^	3.96 ± 0.22^d^

*Note*: All values are represented as mean ± SD. Means with different letters are significantly different (*p* < .05).

## 4 DISCUSSION

The incidence of diabetes mellitus type II increased worldwide recently. Various factors could be incriminated like increased aflatoxin exposure which became more likely due to the climatic changes. Several attempts can be made to manage diabetes and lower the toxic effects of aflatoxin among which is the improvement of lifestyle and functional diet.

Microgreens have gained a wide repetition as a functional food since they are highly rich in antioxidants. The values recorded from sprouting clover microgreen (18 days old) that is used in the current study were considered the optimum for consumption. CM contained high content of Gardenin (29.11%) which possesses antihyperlipidemic and hepatoprotective activities in nonalcoholic fatty liver disease as it inhibits α‐amylase, glutathione S‐transferase, α‐glycosidase (Alonso‐Castro et al., 2020). Also benzopyrones, which are found at high levels in cinnamon and many fruits and vegetables and exert variable biological activities like antimicrobial, antioxidant, antiinflammatory, and platelet antiaggregation effects, were of high amount in used CM (Miri et al., 2016).

Retinol (Vitamin A) and its derivative retinoic acid (RA) were present in high amounts in CM. They are crucial in the regulation of epithelial cell growth and cellular differentiation. Retinoid is necessary for vision and RA affects the pattern formation in developing and regenerating limbs and prevents the growth of some malignant cells (Zhu, 2019).

Aflatoxin exposure in the current study decreased body weight and impaired growth which was also reported previously (Knipstein et al., 2015). Moreover, STZ injection resulted in a decreased body weight as recorded before (Guo et al., 2018). Nevertheless, CM feed improved the body weight gain.

The STZ injection induces mild dysfunction in beta cells with a decrease in insulin secretion and increases the blood sugar level (Akinlade et al., 2021; Guo et al., 2018). In the groups fed CM in this study, the insulin and glucose level was improved. Red clover was reported before to have the potential of lowering the blood sugar level. This was attributed to its rich content of Formononetin‐an α‐glucosidase inhibitor (Masuda et al., 2021).

CM was able to decrease insulin resistance in treated groups similar to what was documented in a previous study (Oza & Kulkarni, 2020). This can be attributed to the isoflavone content of red clover which is known to possess an antidiabetic potential (Harini et al., 2012; Oza & Kulkarni, 2020). The intake of clover in the present study also improved the insulin sensitivity. A previous study reported the activation of PPARγ‐regulated genes in liver due to intake of red clover extract in diabetic rats. This leads to the activation of PPARγ which in turn enhances insulin sensitivity and preserves the blood glucose level (Guo & Tabrizchi, 2006; Qiu et al., 2012).

Aflatoxin is a potent hepatoxic and hepatocarcinogenic compound which would in turn elevate liver parameters and hinder liver function (Saad‐Hussein et al., 2021). Furthermore, diabetes results in a disturbance in liver homeostasis with the subsequent elevation of liver enzymes (Islam et al., 2020). Therefore, in the current study, the groups which were diabetic and/or received aflatoxin had an elevated liver function parameters. However, the intake of clover enhanced the liver function parameters. The positive effect of clover on the liver of diabetic mice was similarly reported previously (Qiu et al., 2012).

Streptozotocin causes an elevation of serum creatinine, and blood urea nitrogen similar to our findings (Ashraf et al., 2013). Diabetes also induces an elevation in lactate dehydrogenase as reported in previous study (Malicka et al., 2016). Feeding CM in G4 was able to decrease the creatinine concentration compared to G3 and lowered the LDH activity. This could be related to the antidiabetic potential of CM and its ability to control the blood glucose level and lowering the adverse effect of diabetes (Harini et al., 2012).

The injection with streptozotocin results in histopathological alteration of the islet cells inducing diabetes (Abdel‐Mobdy et al., 2021). The intake of functional food that can protect the beta cells of islets and in turn control diabetes was reported previously (Mohamed et al., 2022). In the present study, clover was able to counteract the toxic effect of aflatoxin and STZ on beta cells.

The area % of insulin‐positive beta cells was moderately decreased in aflatoxin group and greatly in STZ group similar to previous findings (Abdel‐Mobdy et al., 2021; Mohamed et al., 2022). However, it was improved in CM‐fed groups. This indicates that clover was able to maintain the beta cells in islets of Langerhans.

The adverse effect of aflatoxin on liver and kidney histopathology was likewise to previous findings (Abdel‐Latif et al., 2017; Mohamed et al., 2022). Hyperglycemia in diabetes induces adverse effects in the kidney as demonstrated in the current study and previous study (Mohamed et al., 2022). Clover improved the histopathological findings in the treated group which could be related to its content of antioxidants and its antidiabetic potential as revealed by previous studies (Harini et al., 2012; Oza & Kulkarni, 2020).

In the present study, aflatoxin and/or STZ induced oxidative stress similar to previous findings (El‐Nekeety et al., 2014; Lucchesi et al., 2013). The red clover flavonoids inhibited oxidative stress similar to our findings which showed the antioxidant potential of clover (Khorasani Esmaeili et al., 2015).

In conclusion, the feeding of clover microgreen lowered the glucose level of diabetic rats, decreased insulin resistance, improved insulin sensitivity, improved liver and kidney function, improved pancreas, liver and kidney histopathology, and insulin immunohistochemistry of pancreas.

## AUTHOR CONTRIBUTIONS

**Tahany A. A. Aly:** supervision (equal); writing – original draft (equal). **Sara M. Mohamed:** Conceptualization (equal). **Marwa S. Khattab:** methodology, writing‐ reviewing & editing (equal). **Ahmed M. M. Abido:** Writing – original draft (equal). **Emam A. Abdelrahman:** supervision. **Frederick Sarpong:** Validation (equal). **Ammar Al‐Farga**: writing‐reviewing & editing. **Faisal Aqlan:** visualization

## FUNDING INFORMATION

This research received no specific grant from any funding agency in the public, commercial, or not‐for‐profit sectors.

## CONFLICT OF INTEREST

The authors declared no potential conflicts of interest concerning the research, authorship, and/or publication of this article.

## ETHICS STATEMENT

This study was approved by the Institutional Animal Care and Use Committee, Faculty of Veterinary Medicine, Cairo University (Vet.CU.IACUC) (Vet CU01102020224) and was carried out following the guidelines of Care and Use of Laboratory Animals stated by the National Institutes of Health, USPHS.

## DISCLOSE PREPRINT SERVER

We disclose that it is not submitted to a preprint.

## CONSENT TO PARTICIPATE

All have participated and approved the manuscript and agree with submission.

## CONSENT TO PUBLISH

This manuscript has not been published elsewhere and is not under consideration by another journal. We believe that the findings of this study are relevant to the scope of your journal and will be of interest to its readership.

## Data Availability

All raw data are available as supplementary file.
